# Myeloid Mineralocorticoid Receptor During Experimental Ischemic Stroke: Effects of Model and Sex

**DOI:** 10.1161/JAHA.112.002584

**Published:** 2012-10-25

**Authors:** Ryan A. Frieler, Jessica J. Ray, He Meng, Sai P. Ramnarayanan, Michael G. Usher, Enming J. Su, Stefan Berger, David J. Pinsky, Daniel A. Lawrence, Michael M. Wang, Richard M. Mortensen

**Affiliations:** Department of Pharmacology, University of Michigan Medical School, Ann Arbor, MI (R.A.F., R.M.M.); Department of Molecular and Integrative Physiology, University of Michigan Medical School, Ann Arbor, MI (R.A.F., S.P.R., M.G.U., D.J.P., M.M.W., R.M.M.); Department of Internal Medicine, Division of Cardiovascular Medicine, University of Michigan Medical School, Ann Arbor, MI (J.J.R., E.J.S., D.J.P., D.A.L.); Department of Neurology, University of Michigan Medical School, Ann Arbor, MI (H.M., M.M.W.); Department of Internal Medicine, Division of Metabolism, Endocrinology, and Diabetes, University of Michigan Medical School, Ann Arbor, MI (R.M.M.); Department of Medicine, University of Medicine and Dentistry of New JerseyPiscataway, NJ (M.G.U.); German Cancer Research Center (DKFZ), Division Molecular Biology of the Cell IHeidelberg, Germany (S.B.); Department of Neurology, Veterans Administration Ann Arbor Healthcare SystemAnn Arbor, MI (M.M.W.)

**Keywords:** brain ischemia, reperfusion, stroke

## Abstract

**Background:**

Mineralocorticoid receptor (MR) antagonists have protective effects in the brain during experimental ischemic stroke, and we have previously demonstrated a key role for myeloid MR during stroke pathogenesis. In this study, we explore both model- and sex-specific actions of myeloid MR during ischemic stroke.

**Methods and Results:**

The MR antagonist eplerenone significantly reduced the infarct size in male (control, 99.5 mm^3^; eplerenone, 74.2 mm^3^; n=8 to 12 per group) but not female (control, 84.0 mm^3^; eplerenone, 83.7 mm^3^; n=6 to 7 per group) mice after transient (90-minute) middle cerebral artery occlusion. In contrast to MR antagonism, genetic ablation of myeloid MR in female mice significantly reduced infarct size (myeloid MR knockout, 9.4 mm^3^ [5.4 to 36.6]; control, 66.0 mm^3^ [50.0 to 81.4]; n=6 per group) after transient middle cerebral artery occlusion. This was accompanied by reductions in inflammatory gene expression and improvement in neurological function. In contrast to ischemia-reperfusion, myeloid MR–knockout mice were not protected from permanent middle cerebral artery occlusion. The infarct size and inflammatory response after permanent occlusion showed no evidence of protection by myeloid MR knockout in photothrombotic and intraluminal filament models of permanent occlusion.

**Conclusions:**

These studies demonstrate that MR antagonism is protective in male but not female mice during transient middle cerebral artery occlusion, whereas genetic ablation of myeloid MR is protective in both male and female mice. They also highlight important mechanistic differences in the role of myeloid cells in different models of stroke and confirm that specific myeloid phenotypes play key roles in stroke protection.

## Introduction

Mineralocorticoid receptor (MR) activation is a contributing factor in the pathophysiology of a wide range of diseases. Elevated levels of aldosterone, a physiological MR activator, are known to induce hypertension, alter inflammation and fibrosis, and exacerbate cardiovascular diseases. Clinical, therapeutic interventions for the treatment of hypertension, heart failure, and post–myocardial infarction remodeling have successfully used MR antagonists.^1,2^ However, the benefit of this class of drugs could extend to the treatment of other cardiovascular diseases, like ischemic stroke. In fact, the MR antagonists eplerenone and spironolactone both are markedly effective in reducing infarct size and neurological deficit after ischemic stroke in male rats and mice.^3,4,5^

Despite the known protective effects of MR antagonists, the exact mechanisms of protection are not well understood. In addition to demonstrating a remarkable efficacy of MR antagonists to decrease risk of death during heart failure, the Randomized Aldactone Evaluation Study (RALES) study also showed that these effects occur without altering blood pressure.^2^ Furthermore, spironolactone and eplerenone protected rodents from stroke injury without affecting blood pressure.^3,5^ However, new information recently has come to light as a result of cell-specific genetic ablation techniques that allow for localization of MR activity key to the pathophysiology of disease.

We have previously identified MR as a regulator of macrophage activation and demonstrated that MR antagonists or myeloid MR knockout (MyMRKO) induces an alternatively activated macrophage (AAM) phenotype.^6^ Conversely, MR activation by aldosterone enhances a proinflammatory, classically activated macrophage (CAM) phenotype. Macrophage phenotypes influence the outcome in cardiac remodeling after infarct,^7,8^ and deletion of MR from myeloid cells reduced cardiac remodeling due to *N*^G^-nitro-l-arginine methyl ester/angiotensin II.^6^ These studies demonstrated that MR activation in myeloid cells plays a key role in the promotion of pathophysiological cardiac remodeling.

The neuroprotective effects of MR antagonists have become the focus of many recent studies, and it is now known that MR activation in myeloid cells plays an important role during ischemic stroke. MyMRKO mice are protected from ischemia-reperfusion injury in the brain, which demonstrates the importance of myeloid cells as targets for MR antagonists during ischemic stroke.^9^ Previous studies also indicated that female rats lack responsiveness to MR antagonists^10^; therefore, we tested whether genetic myeloid MR ablation was protective in females during ischemic stroke. To further evaluate and characterize this neuroprotective phenotype, we also tested whether MyMRKO mice were protected in models of permanent middle cerebral artery (MCA) occlusion.

## Methods

### Mice

All animal procedures were performed in accordance with the Guide for the Care and Use of Laboratory Animals (NIH Publication No. 80-23) and were approved by the University Committee on Use and Care of Animals of the University of Michigan. Adult male and female mice weighing between 25 and 30 g were used. MyMRKO mice (MR^flox/flox^; LysMCre^+/−^) and littermate controls (MR^flox/flox^) on a C57BL/6 background were generated as described previously.^6^ Wild-type C57BL/6J mice were purchased from The Jackson Laboratory (Bar Harbor, ME, USA), and MyMRKO and FC mice were bred in-house. Mice were maintained on standard laboratory chow (5001, LabDiet) and water ad libitum. Mice were administered eplerenone (Sandoz, Princeton, NJ) (160 mg/kg per day) in rodent chow (Harlan Teklad, TD.10030) for 1 week before experiments.

### Intraluminal Filament MCA Occlusion

MCA occlusion by the intraluminal filament method was performed as previously described.^11^ The mice were anesthetized with 1% to 3% isoflurane, and a 6-0 silicon rubber–coated nylon monofilament (Docoll Corporation, CA) was inserted into the right internal carotid artery. Regional cerebral blood flow was monitored by laser Doppler flowmetry (Transonic BLF21) before and during monofilament insertion to verify MCA occlusion. Occlusion was defined as a reduction in cerebral blood flow to a level <20% of baseline. For ischemia-reperfusion studies, the suture was removed after 90 minutes, and animals were euthanized 24 hours after suture removal. For permanent occlusion experiments, the suture remained tied in place until the animals were euthanized.

### Photothrombotic Permanent MCA Occlusion

For induction of photothrombotic stroke, the temporalis muscle was transected, and the left MCA was exposed by drilling a 1-mm burr hole through the skull. A laser Doppler flow probe (Type N [18 Ga], Transonic Systems) was placed distal to the exposed MCA to monitor cerebral blood flow. A 3.5-mW green light laser (540 nm, Melles Griot) was directed at the MCA, and Rose Bengal stain (Acros Organics) was injected intravenously (50 mg/kg). The relative tissue perfusion units of the cerebral cortex were monitored continuously with a laser Doppler flowmeter (Transonic BLF21). Stable occlusion was defined as a drop in tissue perfusion units to a level <20% of baseline for >10 minutes.

### Measurement of Infarct Volume

All infarcts were analyzed with magnetic resonance imaging (MRI). At 24 and 72 hours after transient or permanent MCA occlusion, mice were anesthetized with 2% isoflurane/air mixture throughout MRI examination. Mice lay prone, head first in a 7.0T Varian Unity Inova MR scanner (183-mm horizontal bore; Varian, Palo Alto, CA), with the body temperature maintained at 37°C by forced heated air. A double-tuned volume radiofrequency coil was used to scan the head region of the mice. Axial T2-weighted images were acquired through the use of a fast spin-echo sequence with the following parameters: repetition time/effective echo time, 4000/60 ms; echo spacing, 15 ms; number of echoes, 8; field of view, 20×20 mm; matrix, 256×128; slice thickness, 0.5 mm; number of slices, 25; and number of scans, 1 (total scan time ≍2.5 minutes). The infarct volumes were analyzed in NIH ImageJ software (version 1.43) by a blinded observer, and infarct volumes were corrected to account for brain swelling.^12,13^ The following equation was used to calculate the corrected T2-lesion volumes:

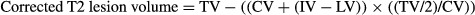

TV indicates total volume in both hemispheres; CV, contralateral volume; IV, ipsilateral volume; and LV, lesion volume.

### Evaluation of Neurological Deficit

Neurological deficits were determined 24 hours after MCA occlusion. Neurological scores were assigned on the basis of the following criteria: 0, no deficit; 1, forelimb flexion and torso turning to the contralateral side when held by tail; 2, circling to contralateral side; 3, unable to bear weight on contralateral side; and 4, no spontaneous locomotor activity.

### Gene Expression Analysis

mRNA expression was measured with quantitative reverse transcription–polymerase chain reaction. Total RNA was extracted from frozen whole cerebral hemispheres through the use of TRIzol reagent and then was purified with the RNeasy Mini Kit (Qiagen). Purified RNA (1 μg) was reverse transcribed to cDNA with an Applied Biosystems kit. Quantitative reverse transcription–polymerase chain reaction was performed with a Bio-Rad iCycler. The relative mRNA expression was quantified by the comparative method, and mRNA was normalized to β-actin.

### Immunohistochemistry

Mice were euthanized and transcardially perfused with heparinized saline (1 U/mL) and then 4% paraformaldehyde. The brains were removed and postfixed in 4% paraformaldehyde for 1 week. The cerebrum then was cut into 2-mm-thick serial coronal sections. Sections were embedded in paraffin, cut into 10-μm sections, and mounted on a slide. Iba1^+^ microglia and macrophages were detected through the use of goat polyclonal anti-Iba1antibody (Abcam, ab5076, Cambridge, MA, USA) at a 1:300 dilution on paraffin-embedded sections. Immunoreactivity was visualized with an ABC kit (Vector Labs, Burlingame, CA, USA) through the use of a biotinlyated rabbit anti-goat secondary antibody and diaminobenzadine. Iba1^+^ cells were quantified and expressed as number of cells per field (40× objective). Two 40× fields were counted by a blinded observer in each anatomic region and were averaged to obtain the number of Iba1^+^ microglia and macrophages.

### Statistics

A Kolmogorov-Smirnov test and normal quantile plots were used to determine if data were normally distributed. For normally distributed data, statistical comparison of mean values between groups was performed with the Student *t* test, and values are presented as mean with standard error of the mean. Data that were not normally distributed were analyzed with the nonparametric Mann-Whitney test and are presented as median with interquartile range. All statistical analysis of data was performed in GraphPad Prism (version 5; GraphPad Software, Inc). *P*<0.05 was considered significant. A limitation of the present study is its restricted ability to detect small differences between groups because of low statistical power.

## Results

### Female MyMRKO Mice Are Protected From Transient MCA Occlusion

**Figure 1.**
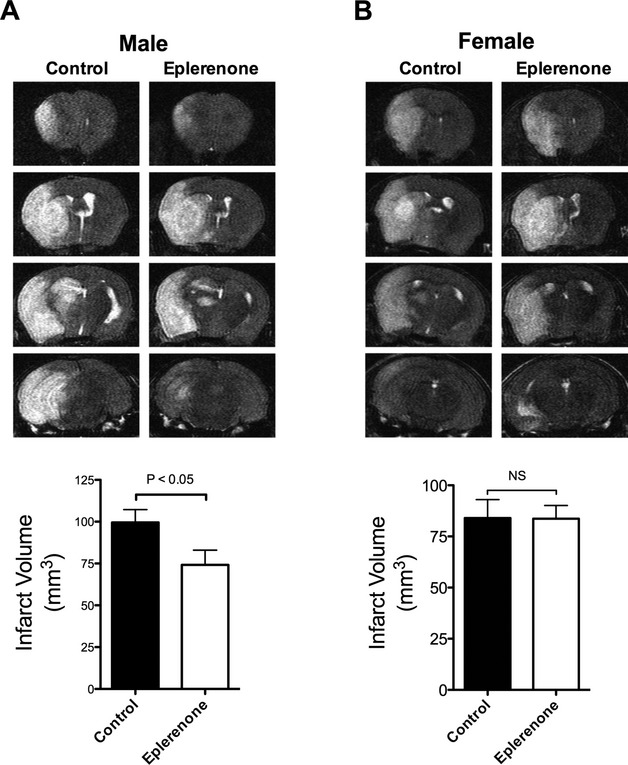
MR antagonism with eplerenone is protective in male but not female mice during transient MCA occlusion. Representative MRI sections and infarct volumes from control and eplerenone-treated (A) male and (B) female mice 24 h after transient (90-minute) MCA occlusion. Data are expressed as mean with standard error of the mean. n=6 to 12 per group.

**Figure 2.**
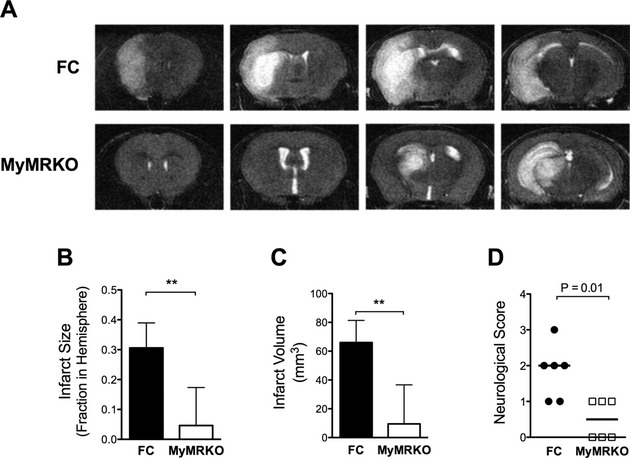
Infarct size and neurological deficit in female MyMRKO mice after transient MCA occlusion. A, Representative MRI sections from FC and MyMRKO mice 24 h after transient (90-minute) MCA occlusion. Infarct sizes in FC and MyMRKO mice represented as (B) fraction in ipsilateral hemisphere and (C) total infarct volume. D, Functional outcome was assessed by scoring neurological deficit in mice after 24 h. Data are expressed as median with interquartile range. n=6 per group.

Previous studies indicated that MR antagonists are protective in male but not female stroke-prone spontaneously hypertensive rats during ischemic stroke.^10^ Similarly, we found that female mice also lack responsiveness to the MR antagonist eplerenone during 90-minute transient MCA occlusion followed by 24 hours of reperfusion. Pretreatment with eplerenone reduced the infarct size in male mice (control, 99.5 mm^3^; eplerenone, 74.2 mm^3^). In female mice, the eplerenone-treated group showed no important decrease in infarct size (control, 84.0 mm^3^; eplerenone, 83.7 mm^3^) (Figure 1A and 1B). Male MyMRKO mice are highly protected during ischemia-reperfusion in the brain.^9^ To determine whether a sexual dimorphic effect also exists in genetically ablated MyMRKO mice, we subjected female mice to transient MCA occlusion. We performed a 90-minute transient MCA occlusion followed by 24 hours of reperfusion, and the infarcts were assessed by T2-weighted MRI. Analysis of MRI scans showed a dramatic reduction in infarct size in female MyMRKO mice (Figure 2A). This is consistent with what we have observed in male mice. Quantification of infarct sizes in ImageJ software revealed a dramatic reduction in infarct size in the ischemic hemisphere of MyMRKO compared to floxed controls (FC) (Figure 2B). The median (interquartile range) was 66.0 mm^3^ (50.0 to 81.4) in the FC group and 9.4 mm^3^ (5.4 to 36.6) in the MyMRKO group (*P*=0.004) (Figure 2C). The reduction in infarct size in MyMRKO mice was accompanied by an improvement in neurological function, indicated by lower neurological scores (median [interquartile range]: FC, 2.0 [1.0 to 2.3]; MyMRKO, 0.5 [0 to 1.0]; *P=*0.01) (Figure 2D).

### Female MyMRKO Mice Have a Suppressed Inflammatory Response

**Figure 3.**
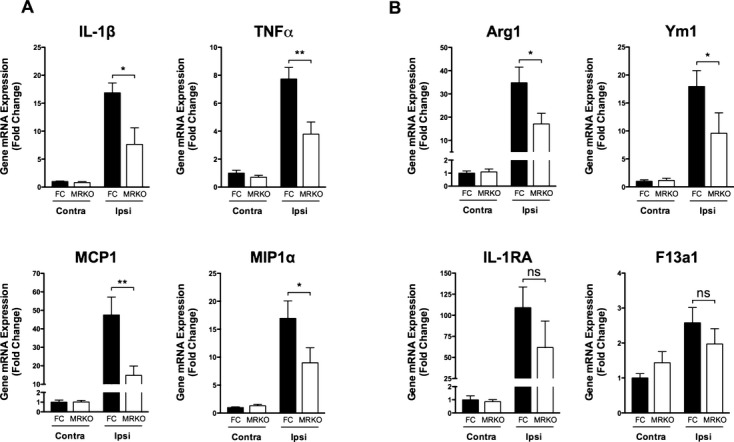
Female MyMRKO mice have suppressed inflammatory gene expression after transient MCA occlusion. Gene expression of (A) proinflammatory CAM markers and (B) AAM markers 24 h after transient (90-minute) MCA occlusion. All genes were normalized to β-actin. *P<0.05, **P<0.01. Data are expressed as mean with standard error of the mean. n=6 per group. Contra indicates contralateral; Ipsi, ipsilateral.

Because MR is a regulator of macrophage polarization, we analyzed the mRNA expression of genes induced in CAM and AAM phenotypes by using quantitative reverse transcription–polymerase chain reaction. Female MyMRKO mice exposed to transient MCA occlusion exhibited a dramatic suppression in inflammatory gene expression compared to similarly treated FC female mice. The expression of proinflammatory CAM genes *(IL-1β, TNF-α, MCP1,* and *MIP1α)* was suppressed in ischemic hemispheres of MyMRKO mice as compared with FC mice (Figure 3A). We also examined a panel of genes *(Arg1, Ym1, IL1RA,* and *F13a1)* expressed by AAMs. The expression of *Arg1* and *YM1* was suppressed in MyMRKO mice (Figure 3B). No statistically significant differences in *IL1RA* and *F13a1* were detected between MyMRKO and FC mice, but their average levels of expression were lower, and this study is limited by low power.

### MyMRKO Mice Are Not Protected During Permanent MCA Occlusion

**Figure 4.**
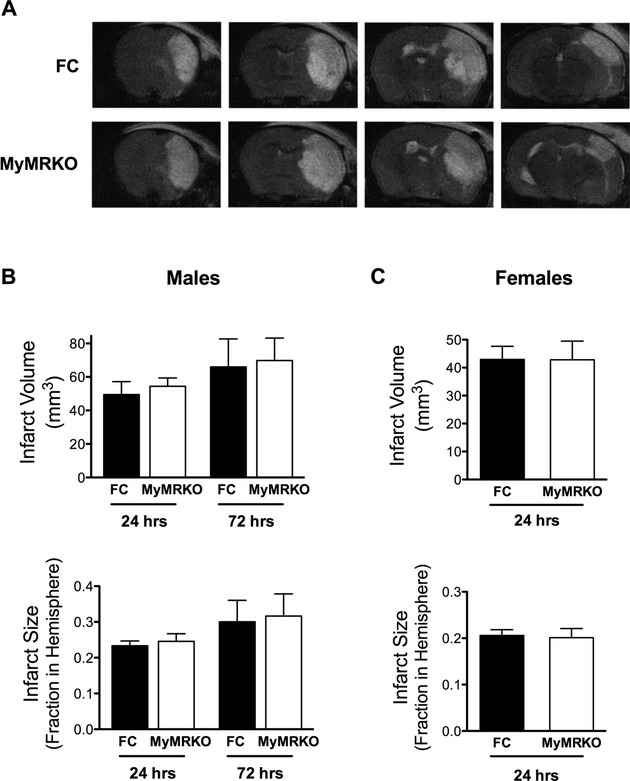
Infarct size in MyMRKO mice after permanent, photothrombotic MCA occlusion. A, Representative MRI sections from male MyMRKO mice 24 h after MCA photothrombosis. Infarct sizes in (B) male and (C) female FC and MyMRKO mice represented as fraction in ipsilateral hemisphere and total infarct volume. Data are expressed as median with interquartile range. n=5 to 6 per group.

MR antagonists have been shown to provide neuroprotection during both transient and permanent occlusion models of ischemic stroke. To evaluate the role of MyMRKO in conditions of permanent occlusion, we subjected MyMRKO and FC mice to MCA photothrombosis. Infarct sizes were assessed by MRI 24 hours after MCA photothrombosis. Surprisingly, no important differences in infarct volumes were observed between MyMRKO and controls among both male and female mice (Figure 4). Because a reduction in perfusion during permanent occlusion reduces infiltration of circulating inflammatory cells, we also assessed the infarcts at 72 hours, when more peripheral immune cells are recruited.^14^ Larger infarct volumes were observed at 72 hours because of infarct expansion, but there continued to be no protection by MyMRKO.

**Figure 5.**
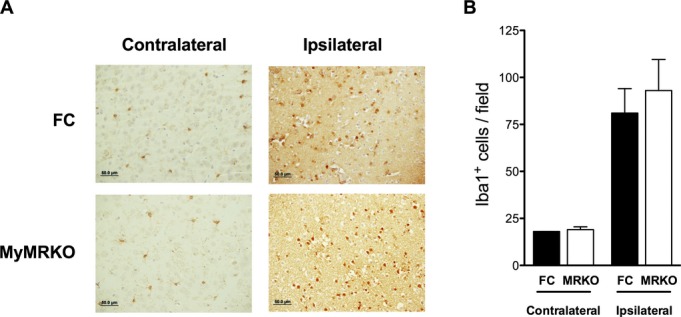
Immunohistochemical analysis of macrophages and microglia 24 h after photothrombotic MCA occlusion. A, Representative photomicrographs of contralateral and ipsilateral hemispheres from FC and MyMRKO mice stained with microglia- and macrophage-immunoreactive Iba1 antibody. B, Quantification of Iba1 immunoreactive cells in the infarct core. Data are expressed as median with interquartile range. n=5 per group.

**Figure 6.**
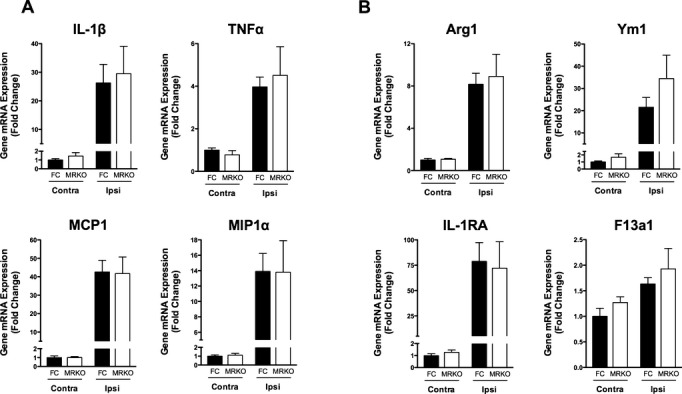
Expression of inflammatory markers in FC and MyMRKO mice 24 h after photothrombotic MCA occlusion. Gene expression of (A) proinflammatory CAM markers and (B) AAM markers 24 h after MCA photothrombosis. All genes were normalized to β-actin. Data are expressed as mean with standard error of the mean. n=6 per group. Contra indicates contralateral; Ipsi, ipsilateral.

We had previously observed a reduction in the number of Iba1+ cells (microglia and macrophages) in the infarct core in MyMRKO mice during transient occlusion.^9^ Therefore, we examined whether there were changes in microglia and macrophage numbers during permanent MCA photothrombosis. Increases in Iba1^+^ microglia and macrophages were present in the ipsilateral hemisphere of both MyMRKO and FC mice, but no differences were detected between MyMRKO and FC mice (Figure 5). Similarly, no baseline differences were observed in the contralateral hemispheres. To further evaluate the changes in the inflammatory response, we analyzed the CAM and AAM markers. The gene expression of CAM markers *(IL-1β, TNF-α, MCP1,* and *MIP1α)* were all significantly increased in the ipsilateral hemisphere of MyMRKO and FC mice compared to the contralateral hemisphere, but no changes between groups were observed (Figure 6A). Similarly, no significant differences were detected in AAM markers between MyMRKO and FC mice (Figure 6B).

**Figure 7.**
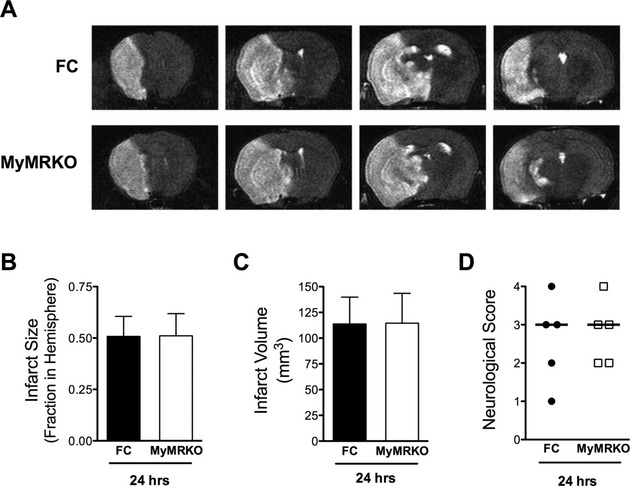
Infarct size in MyMRKO mice after permanent MCA occlusion with the intraluminal filament model. A, Representative MRI sections from male FC and MyMRKO mice 24 h after permanent MCA occlusion. Infarct sizes in FC and MyMRKO mice represented as (B) fraction in ipsilateral hemisphere and (C) total infarct volume. D, Functional outcome was assessed by scoring neurological deficit in mice after 24 h. Data are expressed as median with interquartile range. n=5 per group.

MR antagonists have not been tested in the photothrombosis stroke model, and it is unknown whether they are protective in this model. Therefore, we also tested the effect of MyMRKO during permanent MCA occlusion by using the intraluminal filament model, in which the MR antagonists are known to be effective at reducing infarct size.^3^ Consistent with the results from MCA photothrombosis, we found no protection by MyMRKO with regard to infarct size or neurological deficit 24 hours after permanent MCA occlusion with the intraluminal filament model (Figure 7). These data indicate that reperfusion is necessary for myeloid cell phenotypes to alter stroke outcome.

## Discussion

In the present study, we evaluated the role of myeloid MR in female mice and in multiple models of ischemic stroke. We found that male but not female mice were protected from cerebral ischemia by pretreatment with the MR antagonist eplerenone. In contrast to MR antagonism, genetic ablation of MR in myeloid cells is neuroprotective in female mice. Like males, female MyMRKO mice were dramatically protected from transient occlusion and had dramatic reductions in infarct size, neurological deficit, and inflammatory response. Furthermore, we also show that the neuroprotective phenotype in MyMRKO mice exists during transient MCA occlusion but not during permanent MCA occlusion. MyMRKO mice displayed no differences in infarct size and inflammation in MCA photothrombosis and intraluminal filament models of permanent occlusion.

It has been well established that MR antagonists have protective effects and reduce stroke lesion volumes in male mice and stroke-prone spontaneously hypertensive rats. The majority of experimental cardiovascular studies in animal models only report the use of male animals. The Stroke Therapy Academic Industry Roundtable (STAIR) recommendations for performing preclinical stroke studies include the use of multiple models (permanent and transient occlusion) in both male and female subjects to better assess the viability of drugs for clinical translation.^15^ In accordance, we investigated the effects of the MR antagonist eplerenone during transient MCA occlusion in both sexes and discovered that it is protective in male but not female mice. Although the statistical power in this study was low, we detected no indication of protection in eplerenone-treated female mice. This is consistent with a previous report published by Rigsby and colleagues^10^ demonstrating that neuroprotection by MR antagonists does not extend to female rats. They found that nonovarectomized and ovarectomized female rats lacked responsiveness to both spironolactone and eplerenone during ischemic stroke. Female stroke-prone spontaneously hypertensive rats also were shown to have elevated levels of MR in the cerebral vasculature as compared to males, but it is not known whether this contributes to the sexual dimorphism of MR antagonists.

MR antagonists also have been shown to display sexual dimorphism in their ability to reduce blood pressure. In a salt-induced hypertension model, the MR antagonist spironolactone was effective in reducing blood pressure in male but not female Wistar rats.^16^ Similarly, intracerebroventricular injection of the MR antagonist RU28318 resulted in a reduced antihypertensive response in female rats compared to males.^17,18^ Despite the differences in the likely mechanism of blood pressure lowering in these 2 models, females lacked responsiveness to MR blockade. The sexual dimorphic effects of MR antagonists have been suggested to be due to alternative drug metabolism in females, and it has been shown that the MR antagonist eplerenone is differentially metabolized in male and female mice. Interestingly, though, male rats have been shown to metabolize eplerenone more rapidly than females, making this explanation unlikely.^19^ Furthermore, this hypothesis is confounded by the observation that MR antagonists have beneficial effects in females during models of cardiac remodeling and cerebral aneurysm formation.^20,21,22^ Taken together, the available data suggest that the sexual dimorphic actions might be due to model-specific effects rather than differential drug metabolism.

In contrast to MR antagonism, our studies show that genetic ablation of myeloid MR is protective in both male and female mice after transient MCA occlusion. MyMRKO reduced infarct volume and suppressed inflammation while improving neurological function. These results indicate that there could be differential sexual dimorphism of MR antagonists in different cell types. In fact, MR clearly has effects in other cell types within the brain and cerebrovasculature, and MR overexpression in neurons actually has been shown to have beneficial effects during cerebral ischemia.^23^ It is possible, then, that sex-dependent differences in the expression and regulation of neuronal MR or in the intracellular metabolism of MR agonists or antagonists could play a role in sexually dimorphic responses to MR antagonists in stroke. The studies by Rahmouni and colleagues^17^ in which intracerebroventricular injections of MR antagonists exhibited reduced efficacy in females might indeed indicate that sexual dimorphism of MR antagonists exists in nonmyeloid cell types in the brain. In light of these data, our studies indicate that myeloid-targeted drug delivery could be an effective strategy in the treatment of stroke. Modification of drugs to reduce passage across the blood–brain barrier could provide a means to target circulating cells without affecting other cell types in the brain.

MR antagonists are protective in both transient and permanent MCA occlusion. In contrast, MyMRKO mice were protected during transient MCA occlusion but not during models of permanent MCA occlusion. There were no important differences detected in infarct size, macrophage/microglia recruitment, and inflammatory gene expression between MyMRKO and controls subjected to photothrombotic stroke. Similarly, no important differences in infarct size or neurological deficit were observed during permanent occlusion with the intraluminal filament model. Our finding that MyMRKO is protective in transient but not permanent occlusion could provide important insight into the mechanism of protection with myeloid-specific MR ablation and the role that myeloid MR plays in the pathophysiology of stroke. Several studies targeting inflammatory molecules (neutrophil elastase, CD11b/CD18, matrix metalloproteinases, intercellular adhesion molecule 1) also have shown protective effects in transient but not permanent occlusion.^24,25,26,27,28^ Our present experiments support the hypothesis that inflammatory cells are a key component of the response to reperfusion injury and also demonstrate a key role for the MR and the macrophage lineage in this process.

In permanent occlusion models of stroke, cerebral blood flow is not restored, and there is reduced and delayed inflammatory cell infiltration from the peripheral circulation. The supply of infiltrating cells from the periphery is dependent on the extent collateral vasculature. Thus, much of the early inflammatory cell recruitment during permanent occlusion is due to resident immune cells within the brain parenchyma. This might suggest that neuroprotection in MyMRKO mice during transient occlusion is a result of modulating the trafficking of circulating myeloid immune cells rather than altering the activation phenotypes of microglia or macrophages within the brain parenchyma. However, temporal changes in macrophage activation states after ischemic stroke are largely unknown, and alterations in the balance of CAM and AAM phenotypes could be a crucial factor in altering stroke severity. Female MyMRKO mice exhibited suppression of both CAM and AAM markers 24 hours after transient ischemia, but it will be important to examine the phenotypic changes in activation states at earlier time points. Suppression of some AAM markers was greater in females than in males (described previously) and could reflect previously observed sexually dimorphic Th1/Th2 immune responses.^29,30,31,32^ Because myeloid cells are important in reperfusion injury and oxidative damage, a reduction in inflammatory cell recruitment or decrease in CAM phenotypes also could reduce reperfusion injury.

In conclusion, we demonstrate that genetic ablation of myeloid MR, but not MR antagonism, is protective during transient occlusion in female mice. Our results further delineate the actions of myeloid MR during ischemic stroke and indicate that myeloid MR plays a more important role in reperfusion injury. Thus, MR-targeted drug development might be a feasible therapeutic intervention for stroke, particularly when combined with reperfusion strategies.
